# The Interplay between Circadian System, Cholesterol Synthesis, and Steroidogenesis Affects Various Aspects of Female Reproduction

**DOI:** 10.3389/fendo.2013.00111

**Published:** 2013-09-02

**Authors:** Ziga Urlep, Damjana Rozman

**Affiliations:** ^1^Center for Functional Genomics and Bio-Chips, Institute for Biochemistry, Faculty of Medicine, University of Ljubljana, Ljubljana, Slovenia

**Keywords:** cholesterol, estrogens, signaling, circadian clocks, steroid hormones, regulation

## Abstract

Circadian aspect of reproduction has gained much attention in recent years. In mammals, it is very important that the timing of greatest sexual motivation is in line with the highest fertility. Peripheral clocks have been found to reside also in reproductive organs, such as the uterus and ovary. The timing signal from the suprachiasmatic nucleus is suggested to be transmitted *via* hormonal and neural mechanisms, and could thus mediate circadian expression of target genes in these organs. In turn, estrogens from the ovary have been found to signal back to the hypothalamus, completing the feedback loop. In this review we will focus on the interplay between clock and estrogens. Estradiol has been directly linked with expression of *Per1* and *Per2* in the uterus. CLOCK, on the other hand, has been shown to alter estradiol signaling. We also present the idea that cholesterol could play a vital role in the regulation of reproduction. Cholesterol synthesis itself is circadially regulated and has been found to interfere with steroidogenesis in the ovary on the molecular level. This review presents a systems view on how the interplay between circadian clock, steroidogenesis, and cholesterol synthesis affect various aspects of mammalian reproduction.

## Introduction

Like other organisms, mammals are adapted to the 24 h environmental day/night cycle *via* an internal clock. These evolutionary conserved rhythms are related to daily and yearly changes due to Earth’s rotation and translation as well as to food availability, social interactions, and increased chances of reproduction and survival. Virtually every mammalian cell contains an autonomous circadian clock. In peripheral tissues they are proposed to drive rhythms of gene transcription and in turn govern daily oscillation of many physiological processes. The mammalian timing system is organized in a hierarchical manner. The central pacemaker resides in the suprachiasmatic nucleus (SCN) in the hypothalamus. SCN neurons receive direct photic input from the retina entraining them to the environmental light and dark cycle (1). In turn, the SCN synchronizes other oscillators in the brain and peripheral tissues through endocrine or neural mechanisms, modulation of body temperature and feeding behavior (2). In mammals, it is very important that the timing of greatest sexual motivation is in line with the highest fertility. Therefore hormonal stimuli governing these aspects of reproduction must be under strict control. The circadian control of these mechanisms has been known for a long time as well as the dependency of the reproductive cycle on estradiol levels (3, 4). The SCN mediates its effects on reproduction through direct and indirect neural projections to the hypothalamic-pituitary-gonadal (HPG) axis (5). In females it is responsible for providing a stimulatory signal for the onset of the preovulatory luteinizing hormone (LH) surge (6). Aside from the SCN, sufficiently high concentrations of estradiol are necessary for the LH surge to begin (7, 8). Estradiol is synthesized mainly by the ovary in response to the stimulation by gonadotropins from the HPG axis. Two proteins have been the center of research regarding its production. The steroidogenic acute regulatory (StAR) protein is responsible for cholesterol transportation to the mitochondrial inner membrane. Aromatase is the final enzyme in estrogen synthesis, converting testosterone to estradiol and androstendione to estrone. The expression of both is induced by gonadotropin stimulation *via* cAMP responsive transcription factors, such as the cAMP response element binding protein (CREB) (9). Estradiol was found to influence the expression of clock genes in peripheral tissues, including the uterus, while clock proteins were found to interfere with estradiol signaling, providing an interplay between both systems (10–13).

In this review we will approach the complex relationships between estrogens and circadian rhythmicity and how this influences the female reproductive cycle. Since most research was done on model organisms, mainly rodents, the nomenclature of genes will be written accordingly, unless otherwise specified.

## Molecular Basis of the Circadian Rhythm

The basic mechanism of the mammalian circadian rhythm is a transcriptional-translational-post-translational autoregulatory feedback loop. The core of the loop consists of *Clock* and *Bmal1*. CLOCK and BMAL1 proteins form a dimer which binds to the E-box region in promoters of period (*Per1*, *Per2*, *Per3*) and cryptochrome (*Cry1*, *Cry2*) genes (14–17). Following transcription and translation, PER, and CRY proteins form a complex with casein kinase 1ɛ and translocate into the nucleus. Here they bind to BMAL1/CLOCK complex and inhibit their own transcription, which completes the basic autoregulatory loop (18). PER and CRY proteins are then tagged for proteasomal degradation *via* phosphorylation by casein kinase 1ɛ and 1δ and subsequently by ubiquitination. This cycle lasts approximately 24 h. BMAL1/CLOCK heterodimer also upregulates the transcription of *Rev-erb*α and *Rora*. Their protein products interact with ROR elements (RORE) in the promoter of *Bmal1* gene, up (RORα) or downregulating (REV-ERBα) its transcription (19, 20).

## Pathways Toward and from the SCN

As a master pacemaker and synchronizer the SCN maintains a near 24 h daily rhythm in all cells, hence the name circadian (“circa” meaning approximately and “diem” meaning day) (21). It is responsible for sensing the time of the day *via* outside cues and transmitting that information to oscillators throughout the body, in order to synchronize and entrain their cycles. Neurons within the SCN are organized in a coupling manner to provide a more robust and precise rhythm than individual cells (22). Light information is detected by cells within the retina. Intrinsically photosensitive retinal ganglion cells (ipRGCs), which contain the photopigment melanopsin, gather data through their intrinsic phototransduction mechanism as well as by extrinsic signals from rods and cones (23–26). This signal is then transmitted *via* the retinohypothalamic tract (RHT) to the core region of the SCN (1, 27). Upon stimulation, RHT neurons release glutamate and pituitary adenylate cyclase-activating polypeptide (PACAP) at synaptic contacts with the SCN (28). This in turn leads to calcium influx into the SCN cells and activation of various kinase pathways [MAPK, CaMK, protein kinase A (PKA)] (29). Kinases phosphorylate and activate CREB, which upon phosphorylation binds to CREB binding protein. This complex then binds to cAMP response elements (CRE) in the promoter regions of target genes, altering their transcription (30). *Per1* and *Per2* are two target genes that are influenced by light. They are activated by light signals only during the night and are involved in phase shifts and clock resetting (31–33). Additionally, it has been shown that the human *PER1* gene can also be activated by extracellular stimulators acting through PKA and PKC pathways, and that this activation is distinct from BMAL1/CLOCK regulation (34, 35).

The SCN needs to transmit its timing signals to all other oscillators throughout the body. This is accomplished by utilizing humoral and neural signaling mechanisms (36). Locomotor activity is supposed to be maintained by peptides, such as vasopressin, transforming growth factor α (TGF-α), prokineticin 2, and cardiotrophin-like cytokine (37–40). There are also several proposed pathways used by the SCN to synchronize and entrain peripheral oscillators, such as signaling by hormones and autonomic neural connections, as well as more indirect ways, as is modulation of body temperature and feeding behavior (41–44).

## From SCN through HPG Axis to Sex Hormones

The circadian system is important for successful reproduction, as it ensures that the period of maximal fertility is in line with highest sexual motivation (4). It influences the maturation of the follicle and ovulation as well as timely and successful mating behavior. These complex events and behavior are coordinated in part by the HPG axis and its hormones. Gonadotropin-releasing hormone (GnRH) is the first hormone in the HPG axis. It is released in a pulsatile fashion from the hypothalamus to the anterior pituitary, where it regulates the release of gonadotropins – LH and follicle-stimulating hormone (FSH). Gonadotropins than travel to the reproductive organs, where they trigger the release of sex steroids. On the day of proestrus there is a high release of GnRH with a subsequent LH surge that triggers ovulation.

But where does the SCN come into the picture? A signal from the SCN is crucial for the initiation of the LH surge and for subsequent ovulation (6, 8). When animals are kept under different light/dark cycles, the LH surge still occurs around the time of activity onset (45). In rats a high release of GnRH from the hypothalamus and a subsequent LH surge occur once every 4–5 days. The administration of barbiturates that prevent signaling within the SCN to rats prior to this event blocks the LH surge and ovulation and delays them for 24 h (3). Additionally, studies on SCN lesions in rats resulted in the absence of ovulation (46, 47). Combined, these studies provide evidence that a signal from the circadian system is crucial for successful ovulation.

The LH surge presents a strong signal by the SCN to the periphery. As previously mentioned, circadian clocks are present in peripheral organs, such as the ovary (48). Both FSH and LH have been shown to induce *Per1* and *Per2* expression in rat granulosa cells (49). The effect is most likely mediated by CREB since the promoters of both genes contain CRE elements (50, 51). The importance of the LH surge on the day of proestrus is that it might provide a resetting signal for clocks in the ovary and is thus involved in their synchronization (49, 52).

As for the mechanism by which the SCN coordinates these events, the SCN signals both directly and indirectly to the GnRH neurons in the medial preoptic area (MPOA) (53). Direct signals are believed to be transmitted *via* vasoactive intestinal peptide (VIP) synthesizing neurons (54). These neurons project from the SCN core to the GnRH neurons that contain the VIP receptor (VIPR2/VPAC_2_) (55). The indirect signals are transmitted by vasopressinergic (AVPergic) cells from the SCN shell to the anteroventral paraventricular nucleus (AVPV) (56, 57). The AVPV contains Kiss1 neurons that form an additional link between the SCN and the GnRH neurons. The Kiss1/Kiss1r system has recently been shown to have a big impact on mammalian reproduction. It has been implicated in the onset of puberty (58), preovulatory LH surge (59), GnRH release, including a positive (60) and a negative feedback (61) by sex steroids.

Prior to the discovery of the Kiss1 system, the gonadotropin inhibitory hormone (GnIH) had been identified in the quail brain (62). Since then it has been found in the brains of many other species, including mammals (63). GnIH acts as a negative regulator of the HPG axis suppressing gonadotropin secretion at the pituitary level (64, 65) as well as inhibiting their synthesis (66). In rodents, the GnIH neuron bodies are present in the dorsomedial hypothalamus (DMH) and project monosynaptically to the GnRH neurons (63, 65). The SCN was found to project to the GnIH cells in hamsters, providing a mechanism for the clock to inhibit the negative effect of GnIH on the HPG axis. The SCN could thus have a dual role in ovulatory control, on one hand stimulating GnRH release and on the other preventing the inhibition by the GnIH (67). The GnIH-immunoreactive cells express estrogen receptor-α (ERα), suggesting a feedback by gonadal estrogens (68). GnIH was also found to affect mice ovary, where it inhibits steroidogenesis probably *via* the inhibition of LH receptor (LHR) as well as StAR and 3β-hydroxysteroid dehydrogenase (3β-HSD) proteins (69).

**Figure 1:**
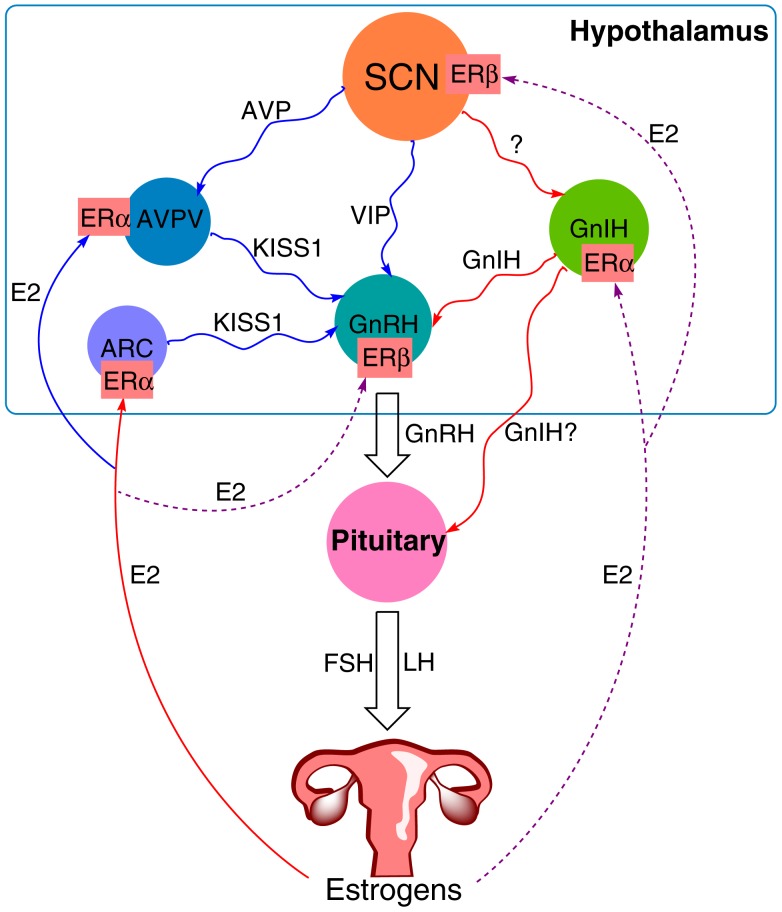
**The proposed model of circadian influence on the HPG axis and feedback by estrogens**. Blue lines depict positive interaction, red lines negative interaction, and purple (solid and dashed) lines depict a complex or not yet known interaction. The SCN signals to various neurons within the hypothalamus, including GnIH, GnRH, and Kiss1 neurons in the AVPV. GnIH neurons negatively regulate GnRH release, whereas the effect of Kiss1 neurons is stimulatory. Estradiol (E2) from the ovary transmits its signals *via* estrogen receptors (ER). It sends negative feedback to Kiss1 neurons in the ARC and positive to Kiss1 neurons in the AVPV. ER are also present on GnRH neurons, GnIH neurons, and the SCN, suggesting a feedback mechanism to these structures as well, although the exact mechanism for these interactions remains yet to be elucidated.

Sex steroids present the final part of the HPG axis. They are synthesized from cholesterol in the gonads in response to gonadotropin stimulation. In females, the ovary is the primary organ for the synthesis of estrogens and progestins apart from the placenta during pregnancy. Estradiol is an important mediator of the positive and negative feedback of GnRH release. During most of the female estrous cycle, low levels of estradiol reduce the amplitude of GnRH pulses (70). Levels of LH are thus kept low while the follicle develops. As the levels of estradiol rise toward the end of the proestrus, the feedback turns to positive with an induction of a high amplitude GnRH surge, followed by the LH surge and ovulation (71). The action of estradiol is mediated through Kiss1 neurons. They are present in the AVPV and the arcuate nucleus (ARC) (72). In contrast to GnRH neurons that express estrogen receptor-β (ERβ), the Kiss1 neurons express mainly ERα, through which estradiol can transmit its signals. The expression of ERβ was also discovered, though its role seems to be less significant since the ovariectomized (OVX) ERβ knockout mice still respond to estradiol stimulation with the upregulation of the *Kiss1* expression, but not the OVX ERα knockout mice. The negative feedback during most of the estrous cycle is a result of estradiol acting on Kiss1 neurons in the ARC, with a following decrease in *Kiss1* mRNA (73). On the other hand, positive feedback on the day of proestrus is a result of the action of estradiol on Kiss1 neurons in the AVPV, where it increases *Kiss1* expression (74). GnIH neurons also express ERα, through which estradiol can exert its influence, though the exact mechanism of this interaction remains to be elucidated. Together these studies show that apart from the signal by the circadian system, estradiol plays a vital part in the induction of the LH surge and triggering of ovulation (Figure 1).

## Disruption of Clock Genes and Impact on Estrous Cycle and Fertility

The SCN itself also contains estrogen receptors, mainly ERβ (75, 76). This enables it to detect plasma estrogen concentrations and react to them accordingly. An increase in estradiol levels during follicular development promotes the formation of synapses between the SCN and GnRH neurons (77) and increases the SCN sensitivity to light (78). To further study the role of circadian timing in reproduction and pregnancy, several mouse mutant models were developed (for summary, see Table 1). Female *Clock^Δ19^* mutant mice (79) produce a dysfunctional CLOCK protein that can form a complex with BMAL1, but the dimer fails to initiate transcription through E-box elements in the promoters of target genes (14, 80). These mice lose central and peripheral rhythmicity in constant DD conditions (79). In view of reproduction, they are fertile with some reports of parturition difficulties, irregular estrous cycles, lack of a coordinated LH surge, and have a higher rate of pregnancy failure (81, 82). These effects are somewhat less in *Clock^Δ19^* + MEL mice, which are able to synthesize melatonin due to a different genetic background (82). Mice lacking a functional *Bmal1* gene show a complete loss of rhythmicity in total darkness (83). Despite this, ovulation does still occur even though there is no apparent LH surge. These mice also have a prolonged estrous cycle, but are infertile due to impaired steroidogenesis and low progesterone levels. There is no evident implantation of the embryo, but it can be reinstituted by progesterone supplementation (84, 85). Two other mouse models were developed, lacking either *Per1* or *Per2* gene (17, 86). Young adult female mutant mice (aged 2–6 months) have regular estrous cycles and the same reproductive success as wild-type females. On the other hand, middle-aged female mutant mice (aged 9–12 months) show lower incidence of estrous cyclicity and have a significantly lower reproductive rate as compared to wild-types. Together these results indicate an accelerated reproductive aging as a consequence of *Per* gene disruption (87). Outside the core clock genes, *Vipr2* null mice were also generated. VIP signaling is important for maintaining rhythmicity and synchrony of neurons within the SCN (88). It is also involved in signaling by VIP and PACAP to the GnRH neurons (89). Mice lacking a functional VPAC_2_ receptor showed elongated estrous cycles exacerbated by constant darkness, however pregnancy rates were not affected (90). Taken together, these studies show that the disruption of core clock genes or SCN signaling pathways has an impact on estrous cyclicity and reproduction. The mechanism underlying these changes remains a matter of debate. Many of the studied mouse models retain rhythmicity under LD conditions, yet still exhibit disrupted reproduction. One possible explanation could be that, while being part of the core clock machinery, many of the disrupted or knocked out genes are transcription factors and could alter the expression of genes involved in reproduction. An example of this would be BMAL1, which may directly regulate *StAR* expression (84).

**Table 1:** **A comparison between different mouse knockout models in view of reproduction**.

Mouse model	Hormone levels	Estrous cyclicity	Reproduction status	Reference
*Per1* KO	No data	Prolonged and irregular cycles	Decreased litter size	(87)
*Per2* KO			Higher loss of implanted embryos	
*Clock^Δ19^*	↓ estradiol	Prolonged and irregular cycles	Higher rate of fetal reabsorption	(81 )
	↓ progesterone	Lack of an LH surge	Increased pregnancy failure	
*Bmal1* KO	↓ progesterone	Reduced number of cycles	Low ovulation rate	(85 )
		Prolonged and irregular cycles	Infertile due to poor embryo development	
*Vipr2*^−/−^	No data	Prolonged cycle, exacerbated by constant darkness	No difference in litter size or pregnancy rates	(90)
			Prolonged delivery	
*Cyp19a1*/*Aromatase* KO	↓ estradiol	No LH surge	Disrupted folliculogenesis	(91 )
	↑ LH and FSH	Disrupted cyclicity due to estradiol deprivation	No ovulation	
			Infertile	
*StAR* KO	↓ progesterone	No data	Impaired folliculogenesis and ovulation	(92)

*Phenotypes of the various knockout models show different effects on reproduction of mice. Disruption of core clock genes causes a marked reduction in reproductive success and loss of regular estrous cyclicity. On the other hand, the lack of functional VPAC_2_ receptors results in milder defects. Disruption of sex hormone synthesis shows a marked effect on reproduction*.

## Cholesterol and Estrogen Synthesis and Link to the Clock

Estrogens are female sex hormones important for the development of secondary female sexual characteristics and enabling successful reproduction. They are produced from cholesterol mainly by the ovary following stimulation by FSH. An enzyme from cholesterol synthesis, lanosterol 14α-demethylase (CYP51) (93, 94) was detected in the rat oocytes on the mRNA and protein levels, suggesting the potential of oocytes to synthesize cholesterol *de novo* (95). This has been confirmed in mice where the CYP51 protein was detected in primary mouse oocytes in a stage- and cell type-specific manner, suggesting distinct regulatory pathways for its expression in the oocyte and the surrounding cumulus cells (96). While it has not yet been proven that the oocyte can indeed synthesize cholesterol, sperm cells retain this ability, which was a surprising discovery (97). In sperm, the major role of the cholesterol synthesis pathway might not be to synthesize cholesterol, but to produce meiosis activating sterols (MAS) (98), whose roles have been reviewed recently (99).

The plasma concentrations of cholesterol vary according to the time of the day, which is of great clinical importance for the hyperlipidemia therapy by statins (100, 101). There have been several studies performed on mouse models with disrupted genes from cholesterol synthesis pathway (102) as well as on models with defects in clock components. It has been shown that mutations in the *Clock* gene abolish circadian expression of *Hmgcr*, the regulatory gene in cholesterol synthesis (103). Signaling by cAMP presents an important mechanism for transmitting circadian information. The proximal promoter of *Hmgcr* reveals one CRE element, but the effect on expression of this gene was mild to none when coupled with overexpression of immediate cAMP early repressor (ICER) or CRE modulator (CREMτ). This suggests an indirect signaling mechanism that controls *Hmgcr* expression (104). The promoter analysis of *Cyp51* from the latter part of cholesterol synthesis identified three CRE elements, and both CREMτ and ICER had a significant effect on *Cyp51* expression. These results are in concordance with findings from *Crem* knockout mice, where in the absence of *Crem*, the circadian regulation of *Cyp51* was abolished, while the expression of *Hmgcr* remained circadian (104). ICER is transcribed from the *Crem* gene and is part of the negative loop of cAMP signaling. It is expressed in a circadian manner and is able to repress its own transcription. ICER contributes to attenuation of cAMP signaling. The canonical signaling pathway (Gαs/cAMP/PKA) has been discovered over 20 years ago (105, 106). After the binding of FSH to its receptor (FSHR), Gαs functionally couples with FSHR and in turn activates adenylate cyclase (AC). This leads to cAMP production by AC and subsequent activation of PKA. Activated PKA’s catalytic subunits then phosphorylate various targets within the cytosol or the nucleus (107). Gene transcription regulated by FSH is controlled by translocation of the PKA catalytic subunit to the nucleus, where it phosphorylates and activates CREB. CREB binds to genes that contain CRE regions in their promoters and activates their transcription (108). ICER has also been found to bind to CRE elements in the promoter of the *Per1* gene and to attenuate its transcription in the adrenal gland. It is proposed to act as a noise filter and might be a mechanism for the central clock to mediate the fine tuning of peripheral clocks (51). Together this presents an interesting aspect, whereby ICER could be involved in circadian regulation of cholesterol and sex steroid synthesis as well as in mediating the effect of the central pacemaker to the periphery. Whether this is true for the ovary still needs to be proven.

**Figure 2:**
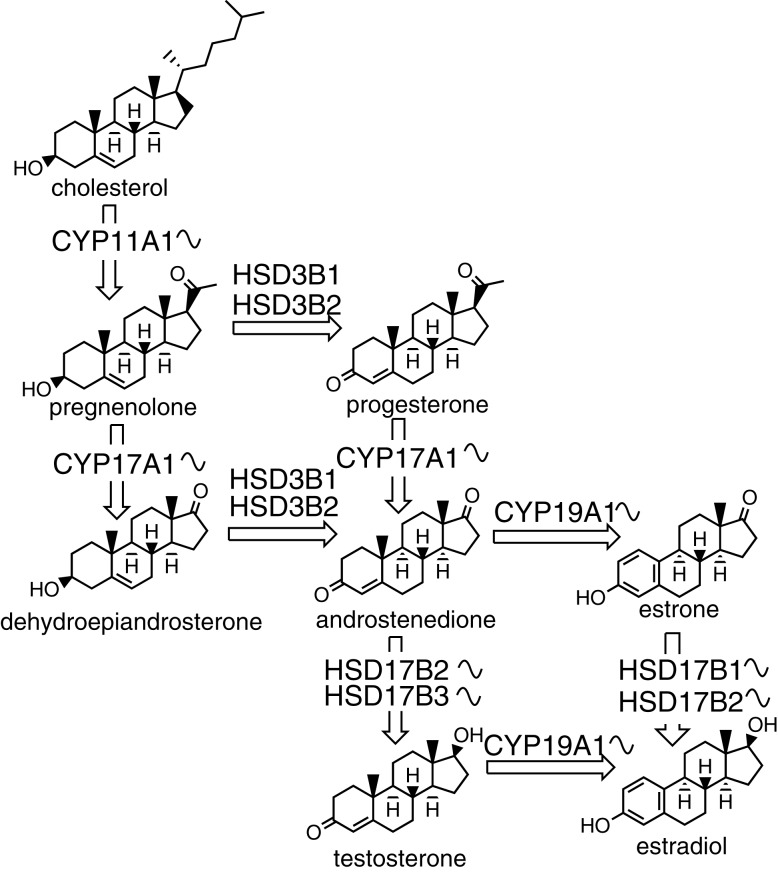
**The biosynthesis of sex steroids**. The sinusoid curve next to an enzyme depicts its known circadian expression. The synthesis starts with cholesterol and consists of several steps catalyzed by either enzymes from the cytochrome P450 or hydroxysteroid dehydrogenase families.

Similar to cholesterol synthesis, cAMP signaling is also important for the production of sex steroids (109). The first step in estrogen synthesis is cleavage of cholesterol by the CYP11A1 forming a C21 product pregnenolone. Pregnenolone presents a branching point in the biosynthetic pathway, as it can be converted to either glucocorticoids and mineralocorticoids or to sex steroids. The CYP17A1 and 3β-HSD then transform pregnenolone to androstenedione. In the next step, androstenedione can be converted by 17β-HSD into testosterone. The final enzyme in the pathway is CYP19A1 (aromatase) that catalyzes the conversion of androstenedione or testosterone by aromatization of the first ring to estrone or estradiol, respectively (Figure 2).

Aromatase encoded by the *Cyp19a1* gene catalyzes the final reaction in estradiol biosynthesis. *Cyp19a1* contains two promoter regions that mediate the effects of FSH through cAMP/PKA pathway: a steroidogenic factor-1 (SF-1) binding site and a CRE like element (9, 110, 111). Apart from the canonical signaling pathway, FSH utilizes various other signaling mechanisms, such as phosphatidylinositol-3 kinase (PI3K) which activates Akt (112). Akt was shown to alleviate the repressive effect of forkhead box O1 (FOXO1) on aromatase expression (113). Additionally, the LH surge induces the expression of the transcription factors CCAAT/enhancer-binding protein β (CEBPB) and ICER (114, 115), both of which are involved in the silencing of *Cyp19a1* (116, 117). In the aromatase knockout mice (ArKO) (118), the females lack endogenous estrogens but have increased levels of testosterone, FSH, and LH. These mice are infertile due to the disruption of folliculogenesis and the lack of corpora lutea (119). The high basal levels of LH and the lack of an LH surge were found responsible for the lack of ovulation (120). An interesting aspect of disrupted estrogen synthesis is the altered sleep-wake rhythms of ArKO mice. They are less nocturnal and are generally less active when compared to WT mice (121, 122).

If we move away from the synthesis itself, we find another crucial protein for the production of sex steroids. The StAR protein is a rate limiting step in steroidogenesis. It is involved in the transport of cholesterol from the outer to the inner mitochondrial membrane. *StAR* knockout mice on steroid replacement therapy show premature ovarian failure with progressive lipid deposition and without detectable corpora lutea (92). The phenotype can in part be explained by insufficient production of progesterone, as progesterone receptor knockout mice exhibit similar defects (123). *StAR* expression and activity in the ovary is regulated by LH *via* cAMP/PKA pathway (124). While the promoter of the human *STAR* gene lacks a consensus CRE sequence (125), CREB, CREM, and activating transcription factor-1 (ATF-1) are still able to bind to it. This is possible by binding to three CRE half-sites identified in the promoter region (126). Several other cAMP responsive transcription factors are involved in *StAR* transcription, such as SF-1, CCAAT/enhancer-binding proteins (CEBPs), sterol regulatory element binding protein (SREBP), and DAX-1 (127–130). Interestingly, the core clock gene *Rev-erb*α induces *StAR* expression in mouse granulosa cells (131). It has been shown that mouse *StAR* promoter region contains putative RORE, which can bind Rev-erbα (132). This indicates that the *StAR* gene might be under the direct control of one of the core clock genes, although there are mixed findings, whether this results in the induction or repression of *StAR* transcription (131, 132). Further research is needed to confirm this hypothesis and to describe the mechanistic background.

## The Influence of Estrogens on the Circadian Rhythm

The effect of steroids on the phase, amplitude, and period of circadian rhythms has been known for a long time (133). This might result from their direct action on the SCN or from one of the previously discussed pathways from the SCN to the periphery. Estradiol shortens the period of *Per2* expression in the uterus of OVX mice, but not in the SCN (11). Estradiol also alters the rhythms of *Per1* and *Per2* in the liver, kidney, and uterus of OVX rats (10). From this we can see that estradiol differentially regulates the expression of clock genes in central and peripheral tissues. The reason for this might in part be due to the differential expression of ERs. As mentioned, the SCN expresses mainly ERβ while liver, kidney, and uterus express predominantly ERα (134, 135). An interesting study by Li et al. directly links the CLOCK protein with ERα activity. Two sumoylation sites were located on the human CLOCK protein and treatment with estradiol promotes this post-translational modification. Sumoylated but not native CLOCK protein was found to modify the transcriptional activity of ERα. This was most likely the result of the direct interaction between sumoylated CLOCK and ERα as concentrations of ERα remained unchanged (13). ERβ was also shown to be directly linked to the core clock genes. The promoter of ERβ contains an evolutionary conserved E-box, which binds core clock proteins to drive rhythmic expression of this receptor. This is in line with the fact that CLOCK/BMAL1 heterodimer has been found to bind to E-box elements in a rhythmic manner (136, 137). These studies provide evidence that there is a complex relationship between estradiol signaling and clock proteins. On one hand, estradiol influences the core clock machinery, and on the other, the core clock proteins may influence ERα transcriptional activity and the expression of ERβ. If and how this affects fertility and reproduction remains to be determined.

## Mevalonate Kinase from Cholesterol Synthesis Interacts with LH Signaling

Cholesterol synthesis is one of the circadially regulated processes (104). In humans cholesterol levels peak toward the morning and are lowest in the afternoon (100). Also, SCN signaling *via* the HPG axis influences in part the production of sex steroids. Both are therefore linked to the central pacemaker, yet little is known whether there is a link connecting them directly. There is one example of the interplay between the two pathways through the regulation of LHR. LHR is expressed in ovarian cells and shows a marked downregulation following the LH surge (138, 139). When LH binds to its receptor, it activates the cAMP/PKA signaling pathway (140). This results in an increase in sex steroid hormone biosynthesis. The increased steroidogenesis leads to cholesterol depletion in the cell, triggering upregulation of genes involved in cholesterol biosynthesis, such as mevalonate kinase (MVK), *via* SREBP (141, 142). MVK catalyzes the conversion of mevalonate to 5-phosphomevalonate in the cholesterol synthesis pathway. Interestingly, MVK also acts as an RNA binding protein. It was found to form a complex with LHR mRNA and prevent its translation, which results in LHR mRNA degradation (143, 144). In the meantime, steroidogenesis is temporarily interrupted until MVK is able to restore its catalytic function.

## Conclusion

With every new publication the importance of biological rhythms in various aspects of our lives becomes more evident. In recent years research has extensively focused on the implications of circadian rhythmicity in reproduction. Mouse knockout models have proven to be invaluable in determining how defects of the clock or impaired steroidogenesis influence reproduction. Interesting to note is that even the defects in functionally related genes, such as *Clock* and *Bmal1*, have different outcomes, with former showing decreased reproduction rates, while the latter proving infertile. In females estrogens present a link between circadian rhythmicity and reproduction. Signals by estradiol and the SCN together are crucial for the start of an LH surge with the resulting ovulation. The neural network within the hypothalamus presents a complex system that integrates both environmental and hormonal signals to ensure successful reproduction. But the relationship between the two doesn’t stop there. It seems that estradiol and circadian signaling pathways are more intimately connected with evidence for their interplay on the molecular level. The core clock genes were found to interfere with estradiol signaling, while estradiol was shown to alter the rhythms of *Per1* and *Per2* gene expression in various tissues. To add another piece to the puzzle, cholesterol synthesis itself is rhythmic, and interferes with steroidogenesis in the ovary *via* the downregulation of LHR.

Despite the complexity of this network of interactions, there is growing evidence that the disruption of one of these systems has an influence on reproduction. But the questions regarding the mechanistic background and the potential application in medicine both require additional research.

## Conflict of Interest Statement

The authors declare that the research was conducted in the absence of any commercial or financial relationships that could be construed as a potential conflict of interest.
